# Enhancing athletic performance: the critical role of oral health in sports

**DOI:** 10.1038/s41432-025-01157-0

**Published:** 2025-05-13

**Authors:** Rawan Kahatab, Ioannis Polyzois

**Affiliations:** School of Dental Science, University of Dublin, Trinity College Dublin, Dublin, Ireland

**Keywords:** Dental diseases, Dental conditions

## Abstract

**A Commentary on:**

**Ferreira R O, Frazão D R, Ferreira M K** **M et al***.*

Periodontal disease and sports performance: a systematic review and meta-analysis. Res Sports Med. 2024; 10.1080/15438627.2023.2235048.

**Data sources:**

A comprehensive search was conducted across PubMed, Scopus, Web of Science, Cochrane Library, and LILACS, supplemented by gray literature searches using OpenGrey and Google Scholar. The search strategy combined MeSH terms and free-text keywords related to “athletes” or “exercise,” and “periodontitis,” “periodontal disease,” or “gingivitis,” along with “sports performance,” adjusted for each database’s syntax

References were managed using EndNote X9, with duplicates removed. Eligibility screening excluded opinion papers, technical articles, guidelines, and animal studies. Full texts of relevant articles were reviewed, and additional articles were identified through citation tracking. Two reviewers independently performed the selection, with disagreements resolved by a third examiner.

**Study selection:**

The study selection in this systematic review involved identifying observational studies that compared professional athletes with periodontal disease (the case group) to those with a healthy periodontium (the control group). The studies included in the review were required to focus on the effects of periodontal disease on sports performance, with no restrictions on the type of sport. Specifically, the researchers developed a PECO statement and conducted searches in online databases for relevant studies published until April 2022, eventually including cross-sectional, case-control, and cohort studies that met their eligibility criteria.

**Data extraction and synthesis:**

For each selected publication, data was collected on various characteristics, including publication year, study design, participant demographics (source and sample size), participants’ ages, periodontal assessment methods, sports performance assessment, and outcomes. If necessary, study authors were contacted via email to obtain missing data.

Quality Assessment: The methodological quality of the studies was evaluated using the Joanna Briggs Institute’s checklist for prevalence studies. Each study was assessed based on criteria such as the clarity of sample inclusion criteria, detailed descriptions of study subjects and settings, validity and reliability of exposure measurements, identification of confounding factors, and appropriateness of statistical analyses.

Data analysis for this study was conducted using RevMan software to determine the relationship between periodontal disease and self-perceived sports performance, measured through Oral Impact on Daily Performance (OIDP) questionnaires; a meta-analysis was conducted using odds ratios (OR) with 95% confidence intervals (CI), comparing the presence of periodontal disease in performance-affected (case) and unaffected (control) groups. A random-effects model was applied when methodological heterogeneity existed, and heterogeneity was assessed via the I² index along with sensitivity analyses to evaluate the impact of individual studies on the overall findings. A funnel plot was generated where appropriate to assess publication bias.

Sensitivity analyses were conducted to validate the influence of individual studies or groups of studies on the pooled results. The overall evidence quality was evaluated using the GRADE approach, which considered factors such as risk of bias, inconsistency, and indirectness.

**Results:**

Out of 793 references identified, 14 were selected for full-text evaluation following the application of eligibility criteria, leading to eight articles being included in the meta-analysis. The studies predominantly included cross-sectional designs and involved athletes whose ages ranged from 16 to 47 years. Various sports were represented, including track and field, boxing, and soccer. Six studies indicated a significant relationship between periodontal disease and self-perceived sports performance, while two studies found no association. The meta-analysis showed a 1.5-fold increased risk of sports performance decline associated with periodontal disease, as measured by the OIDP questionnaire. Specifically, the odds ratio was 1.55 (95% CI: 1.04 to 2.31), indicating with moderate certainty that athletes with periodontal disease are more likely to report a decline in performance.

**Conclusions:**

It can be concluded from this systematic review and meta-analysis that there is a significant association between periodontal disease and reduced self-perceived sports performance in athletes. The findings underscore the importance of maintaining good oral health. Athletes should prioritize periodontal health by engaging in effective oral hygiene practices and seeking timely treatment for periodontal issues to optimize their performance.

## GRADE Rating:



## Commentary

The systematic review conducted by Ferreira et al.^1^ presents a timely and relevant exploration of the association between periodontal disease (PD) and athletic performance. As oral health conditions can impact overall health and well-being, the findings of this review have substantial implications for athletes, coaches, and sports health practitioners.

The authors adhered to the PRISMA guidelines, resulting in a methodologically sound framework for conducting the review. The systematic search across multiple databases, including PubMed and Scopus, enhances the comprehensiveness of the literature included, ensuring that a wide range of studies were evaluated. The use of the PECO framework to structure the review question clearly defines the populations, exposures, comparisons, and outcomes, which is pivotal for readers to grasp the scope of the inquiry.

The statistical analysis conducted in the meta-analysis is another strength, as it allows for the quantification of associations between PD and performance-related outcomes. The moderate association identified between periodontal disease and self-perceived performance reduction provides valuable insight into an area that is often overlooked in sports science. Moreover, the emphasis on athletes implies a targeted understanding of the unique challenges faced by this population concerning oral health.

Nevertheless, the review is not without its limitations. The reliance on observational studies inherently restricts the strength of the conclusions, as these designs are more prone to biases compared to experimental studies. The authors highlighted various quality assessment issues regarding the included studies, such as unclear criteria for participant inclusion and questionable methodological rigor. Such heterogeneity raises concerns about the generalizability of the results and the potential for confounding factors to skew the findings.

While the review aimed to address confounding factors, the complexities inherent in the interaction between periodontal disease and athletic performance make it challenging to establish a clear causal relationship. Factors such as training intensity, dietary habits, and stress levels—often highly individualized among athletes—could interact with both periodontal health and performance outcomes but were not sufficiently controlled in the studies analyzed.

Additionally, while the meta-analysis aimed to synthesize data, the presence of heterogeneity across studies poses a challenge. While low heterogeneity is noted in this systematic review which suggests a degree of agreement across studies, the potential for methodological differences remains critical in evaluating the results and their implications. Further research with standardized methods and comprehensive data collection would help clarify these relationships and reduce inconsistencies.

In conclusion, Ferreira et al.‘s systematic review highlights a possible link between periodontal disease and sports performance. While the review is methodologically strong and uses solid statistical analysis, its conclusions are limited by the quality of the included studies and potential biases. Future research should focus on more robust methods, such as longitudinal studies and randomized controlled trials, to better understand this relationship. A more practical and comprehensive approach that considers a wider range of factors affecting both oral health and athletic performance will be important for making progress in this area.

Practice Points

- Periodontal disease can lead to reductions in training capacity, increased pain, and difficulties in participation which collectively can affect athletic performance.
- Practitioners should encourage athletes to prioritize maintaining good oral hygiene and seek timely treatment for periodontal issues to minimize potential impacts on their athletic performance.
